# Time to diagnosis for breast, cervical and colorectal cancer in Zimbabwe and South Africa: a cross-sectional study

**DOI:** 10.1136/bmjgh-2025-021889

**Published:** 2026-02-11

**Authors:** Jennifer Moodley, Suzanne E Scott, Sarah Day, Bothwell T Guzha, Zvavahera M Chirenje, John E Ataguba, Dharmishta Parmar, Ekaterina Pazukhina, Jonathan Myles, Valerie A Sills, Sudarshan Govender, Fiona M Walter, on behalf of the AWACAN-ED collaborator team, Kakia Anne Faith Namugenyi

**Affiliations:** 1 School of Public Health, University of Cape Town, Rondebosch, South Africa; 2 Wolfson Institute of Population Health, Queen Mary University, London, UK; 3 Department of Obstetrics and Gynaecology, University of Zimbabwe, Harare, Zimbabwe; 4 Bixby Centre for Global Reproductive Health, University of California San Francisco, San Francisco, California, USA; 5 Health Economics Unit, University of Cape Town, Rondebosch, South Africa; 6 Health Economics Laboratory, University of Manitoba, Winnipeg, Manitoba, Canada

**Keywords:** Africa, Africa South of the Sahara, Global Health, Cancer, Cross-sectional survey

## Abstract

**Introduction:**

Shorter time to diagnosis may lead to better cancer outcomes in Southern Africa. This study measured the time from symptoms to first healthcare visit (patient interval; PI) and diagnosis (diagnostic interval; DI) and associated factors for breast, cervical and colorectal cancer in Zimbabwe and South Africa (SA).

**Methods:**

A cross-sectional survey collected data on socio-demographics, cancer awareness, barriers to seeking care, symptoms, healthcare visits and diagnosis after recent cancer diagnosis. Cox regression was used to determine factors associated with PI and DI.

**Results:**

This study included 1021 participants (Zimbabwe 396, SA 625). Symptom and risk factor recall was low. Median PIs were shorter than DIs across cancers and regions. For breast cancer, those reporting more health-seeking barriers had longer PIs (Zimbabwe HR 0.801, 95% CI 0.703 to 0.913; SA HR 0.885, 95% CI 0.817 to 0.958), while greater emotional response to symptoms was associated with a shorter PI (Zimbabwe HR 1.194, 95% CI 1.101 to 1.295; SA HR 1.145, 95% CI 1.079 to 1.216). Interpreting a cervical symptom as serious (Zimbabwe) was associated with a shorter PI. DIs were longer in less-resourced regions and increased with number of healthcare visits before diagnosis. Significantly shorter DIs occurred when the first provider was a clinic doctor or specialist compared with a clinic nurse.

**Conclusions:**

Efforts to improve timely cancer diagnosis in Zimbabwe and SA should focus on supporting primary healthcare providers in managing and referring symptomatic patients, enhancing cancer symptom awareness and interpretation, and addressing barriers to care.

**WHAT IS ALREADY KNOWN ON THIS TOPIC**

**WHAT THIS STUDY ADDS**

- This study provides much-needed high quality data on the time from, and factors associated with symptom awareness to presentation to healthcare (patient interval) and to diagnosis (diagnostic interval) for patients with breast, cervical and colorectal cancer in urban and rural regions in two Southern African countries.
- We found that patients had limited knowledge of cancer symptoms and risk factors, with most experiencing barriers to accessing healthcare. The type of provider (specialist, clinic doctor, clinic nurse) first seen influenced the diagnostic interval.

**HOW THIS STUDY MIGHT AFFECT RESEARCH, PRACTICE OR POLICY**

- In our context, recall of exact dates for onset of symptoms and date of first healthcare provider visit was challenging, despite using calendar landmarking. To address this, we developed a method of using context relevant information (free-text comments) to compute the average interval for use in analyses. This method will need to be tested in future studies.
- This study underscores the need for interventions to (a) support primary healthcare providers in managing and referring patients with possible cancer symptoms and (b) improve public cancer symptom awareness and help-seeking behaviour and address access barriers.

## Introduction

 Cancer is an increasing public health challenge in Southern Africa. In 2022, 1 109 209 new cases and 711 429 deaths were reported.^1^ By 2045, new cases are predicted to increase by 107% and deaths by 91%, placing strain on individuals, communities and healthcare systems.^2^ Rising incidence and mortality are attributed to the high prevalence of risk factors, ageing societies and inequalities in timely care-seeking, provision and quality of care.

Breast, cervical and colorectal cancers (CRCs) are important health concerns in Southern Africa. In 2022, breast and cervical cancer accounted for 25% and 19% of new cancers and 19% and 15% of cancer deaths in the region.^1^ Although breast cancer incidence rates are lower than reported in high-income countries (HICs), mortality rates are similar. Africa has the highest age-standardised incidence rate (ASIR) for cervical cancer (26.4 per 100 000) compared with the global rate (14.1 per 100 000), with Eastern and Southern Africa most affected (ASIRs of 40.4 and 34.9 per 100 000, respectively).^1^ In Africa, CRC is the fifth most commonly diagnosed cancer, with Southern Africa having the highest age-standardised incidence (12.8 per 100 000) and mortality (8.4 per 100 000) rates on the continent.^1^ Factors contributing to the observed higher incidence and mortality rates in Africa compared with rates reported in HICs include: the high HIV prevalence (influencing cervical cancer incidence), low public cancer awareness, late presentation, limited access to healthcare facilities, limited diagnostic capacity and poor access to effective treatment.^3^,^6^

Stage at diagnosis predicts cancer outcomes, with advanced-stage cancer (stages III or IV) being least responsive to curative treatment. In sub-Saharan Africa, most breast (64%), cervical (66%) and colorectal (67%) cancers are diagnosed at an advanced stage.^3^,^5^ Most cancer diagnoses occur after people present to health services with symptoms. Across Africa, cancer screening programmes are limited or ineffective, due to infrastructure requirements, resource constraints and low participation rates. The WHO’s 2020 Report on Cancer recommends prioritising ‘early diagnosis programmes with rapid access to effective treatment’ in low-income and middle-income countries (LMICs), until universal screening programmes are available and equitable. Investment in programmes addressing symptom awareness, timely diagnosis and referral for appropriate treatment is recommended.^6^ International evidence shows that shorter time to diagnosis is associated with better outcomes,^7^ yet little is known about these intervals in Southern Africa. The aim of this study was to determine the time from breast, cervical and CRC symptom awareness to presentation in healthcare and diagnosis, and factors influencing these intervals in two Southern African countries, Zimbabwe and South Africa (SA).

## Methods

### Study design and setting

The aWAreness of CANcer & Early Diagnosis programme (AWACAN-ED) is an NIHR-funded Global Health Research Group that aims to develop and evaluate tools for timely symptomatic diagnosis of cancer and strengthen research capacity in Southern Africa through training programmes and mentorship. ^8^As part of the AWACAN-ED programme, we conducted a cross-sectional survey of patients aged 18 and older recently diagnosed (in the preceding month or within 4 weeks of starting treatment) with breast, cervical or CRC in selected secondary and tertiary public healthcare facilities in Zimbabwe and SA. The two countries represent different economic levels, SA being an upper-income and Zimbabwe a lower-middle-income human development index country.^9^ In each country, two regions with different resource levels were selected. In SA, the Eastern Cape (EC) is more rural and less resourced than the Western Cape (WC) province.^10^ In Zimbabwe, Bulawayo province is less resourced compared with Harare.^11^ Patients with a history of cancer who had started treatment for the newly diagnosed cancer or were unable to provide consent were excluded.

The study was guided by the Model of Pathways to Treatment that identifies key events in the care pathway and patient, disease-related, health system-related and provider-related factors that may contribute to the intervals between these events.^12 13^

### Data collection

Between September 2022 and December 2023, consecutive consenting patients were recruited. Data were collected by trained interviewers using an electronic tablet loaded with the structured questionnaires in local languages. Participants were interviewed in a private room at the health facilities in their preferred language. A pilot study was conducted, and questions were refined for better understanding.

### Measures

Sociodemographic variables included: age, sex, place of residence, highest educational level, employment status, relationship status and household spending on frequently (eg, groceries) and infrequently (eg, education fees) purchased items. The household spending variables were adopted from an existing tool that was previously used in SA.^14^ A 1-month recall period was used to collect information on frequently purchased items, while a 12-month recall period was used for infrequently purchased items. Annual total household expenditure was computed by multiplying monthly spending by 12 and adding to the reported annual expenditure for infrequently purchased items. Per capita expenditure was calculated to reflect the welfare level of each household member. Individuals and households were categorised into socioeconomic quintiles using per capita household expenditure. This was done separately for each country’s dataset. The most deprived household or individual is classified in quintile 1, while the least deprived is in quintile 5.

Information was collected on the history of breast self-examination, clinical breast examination and cervical screening, family history of cancer and a reported history of benign breast or colorectal disease. Awareness of symptoms and risk factors for breast, cervical or CRC was measured using open-ended questions (eg, “Please would you name as many symptoms or signs of breast cancer as you can think of?”), guided by the validated AWACAN breast and cervical cancer and the UK colorectal Cancer Awareness Measure (CAM) tools.^15 16^ Responses were analysed with correctly recognised symptom or risk factors scoring 1 point then summed to compute a total score. Scores were later dichotomised into 0 (no recognition) and ≥1 (recognised at least one symptom/risk factor).

Participants were presented with 16 barriers to seeking care and asked whether they agreed, disagreed or did not know. Barriers were drawn from the AWACAN tool.^15^ Each barrier that the patient agreed with scored 1 and a total was calculated.

We asked participants whether their signs were picked up during a medical visit, a screening test or if they self-presented to a health facility because of symptoms. For those reporting a symptomatic presentation, we asked about their initial symptom and responses were coded based on the AWACAN and colorectal CAM tools. Four questions about initial symptom interpretation were included. Responses were coded Yes scoring 2, No 1 and Unsure 0, with reverse coding where applicable to allow a total score with higher values representing a more serious interpretation of the symptom. Participants’ emotional response to their initial symptom was collected with 4 questions, (response options ‘Yes, very much, Yes, a bit, No, not at all’ and received a score of 2, 1 or 0 respectively). The scores were summed to produce a total score with higher value representing greater emotional response.^17^

With regard to symptoms and journey to care, we asked about the first symptom/change noticed and healthcare provider visits for the symptom and dates for each. We defined the following intervals in alignment with the Model of Pathways to Treatment and the Aarhus Statement^7 13^:

- Patient interval (PI): Time between the initial symptom and first presentation to a healthcare provider.
- Diagnostic interval (DI): Time between the first visit to a healthcare provider and the date of diagnosis.

To minimise recall bias, calendar landmarking^18^ was used to help remember important dates. An approximate time frame was recorded if the patient could not recall the exact date (eg, end of the year). This was used to determine an earliest and a latest possible date for noticing a symptom, and an earliest and latest possible date for first visit to a healthcare provider. The longest and shortest possible PI was then computed and the average used in analyses if the difference between the longest possible PI and shortest possible PI was <2 months. A difference >2 months was deemed to be too inaccurate and participants were excluded from the interval analysis. The same approach was applied to calculating the DI.

Data from medical records included date first seen at referral facility and date of diagnosis (histological and clinical).

### Data analysis

Data were analysed using (R V.4.4.1). Proportions were calculated for categorical and medians and IQRs for continuous variables.

Participant characteristics, symptom and risk factor awareness, symptom interpretations, emotional response and time intervals are reported by country and region. Cox proportional hazards regression was used to determine factors associated with the PI and DI. We first conducted univariate analysis separately for each cancer and each interval using the Wald test. Variables that were significant at p<0.20 in the univariate analysis were included in the initial Cox regression analysis, carried out separately for each cancer, each country and each interval. Separate regression models were fitted as the relationship between some covariates and the outcome (PI and DI) varied considerably by country and cancer type. Pooling all data into a single model would have masked important domain-specific associations and limited our ability to provide more specific tailored recommendations. Due to the low sample size by country for CRC, we fitted a combined country model. Observations with missing values were dropped from the analyses. A backward stepwise procedure was used to reduce the number of covariates at each step leaving only the variables at p<0.10. HRs and 95% CIs are reported.

### Patient and public involvement

The AWACAN-ED programme includes patient and public representatives in the investigator team and research collaborator team.^8^ Patient and public representatives were involved in reviewing the study proposal and data collection instruments. Results of the cross-sectional survey have been discussed with our research collaborator team through a series of workshops, with findings from these workshops contributing to the development of our early diagnosis intervention toolkit.

## Results

A total of 3040 patients were approached to participate in the study, of whom 1117 (36.7%) were eligible (Zimbabwe 404, SA 713). The main reason for ineligibility was a diagnosis occurring more than 1 month prior. Overall, 86 (7.7%) of eligible participants refused participation (Zimbabwe 2.0%, SA 10.9%). A further 10 participants were excluded for other reasons (withdrawals, data lost in upload). The final dataset included 1021 participants (Zimbabwe 396, SA 625).

### Participant profile

Details on participant characteristics by cancer type, country and region are provided in table 1. The median age was 52.6 (IQR 44.3–64.1) for Zimbabwean and 54.0 years (IQR 44.0–64.3) for SA participants. For all cancers, participants in the less-resourced regions were categorised into the lower expenditure quintiles.

**Table 1:** Participant characteristics

Cancer type	Breast						Cervix						Colorectal					
	South Africa			Zimbabwe			South Africa			Zimbabwe			South Africa			Zimbabwe		
Country/region	South Africa N=218	Eastern Cape N=47	Western Cape N=171	Zimbabwe N=99	Harare N=70	Bulawayo N=29	South Africa N=285	Eastern Cape N=166	Western Cape N=119	Zimbabwe N=216	Harare N=142	Bulawayo N=74	South Africa N=122	Eastern Cape N=18	Western Cape N=104	Zimbabwe N=81	Harare N=56	Bulawayo N=25
Sex, n (%)																		
Female	212 (97.3)	45 (96.0)	167 (97.7)	97 (98.0)	68 (97.1)	29 (100)	285 (100)	166 (100)	119 (100)	216 (100)	142 (100)	74 (100)	62 (50.8)	10 (55.6)	52 (50.0)	48 (59.3)	34 (60.7)	14 (56.0)
Male	6 (2.7)	2 (4.0)	4 (2.3)	2 (2.0)	2 (2.9)	0 (0.0)	–	–	–	–	–	–	60 (49.2)	8 (44.4)	52 (50.0)	33 (40.7)	22 (39.3)	11 (44.0)
Age Median (IQR)	57.6 (46.7–66.2)	58.6 (47.4–66.9)	57.4 (46.5–66.2)	52.8 (43.9–64.1)	51.4 (42.0–63.3)	58.7 (50.8–64.1)	49.0 (41.1–58.7)	50.0 (40.9–59.6)	48.0 (41.2–57.0)	51.0 (44.7–61.7)	51.4 (44.2–62.6)	50.9 (44.9–59.6)	61.6 (50.8–70.5)	48.0 (38.2–58.9)	64.0 (54.6–71.7)	58.4 (43.7–67.0)	56.6 (44.1–66.7)	62.5 (41.1–67.7)
Relationship status, n (%)																		
Married or living together with a partner	98 (45.0)	17 (36.2)	81 (47.4)	46 (46.5)	36 (51.4)	10 (34.5)	96 (33.7)	53 (31.9)	43 (36.1)	95 (44.0)	66 (46.5)	29 (39.2)	71 (58.2)	8 (44.4)	63 (60.6)	43 (53.1)	26 (46.4)	17 (68.0)
Single	54 (24.8)	14 (29.8)	40 (23.4)	8 (8.1)	5 (7.1)	3 (10.3)	105 (36.8)	62 (37.4)	43 (36.1)	14 (6.5)	6 (4.2)	8 (10.8)	17 (13.9)	7 (38.9)	10 (9.6)	10 (12.4)	7 (12.5)	3 (11.5)
Separated/ divorced/widowed	66 (30.3)	16 (34.0)	50 (29.2)	45 (45.5)	29 (41.4)	16 (55.2)	84 (29.5)	51 (30.7)	33 (27.7)	106 (49.1)	69 (48.6)	37 (50.0)	34 (27.9)	3 (16.7)	31 (29.8)	28 (34.6)	23 (41.1)	5 (20.0)
Missing	0 (0.0)	0 (0.0)	0 (0.0)	0 (0.0)	0 (0.0)	0 (0.0)	0 (0.0)	0 (0.0)	0 (0.0)	1 (0.5)	1 (0.7)	0 (0.0)	0 (0.0)	0 (0.0)	0 (0.0)	0 (0.0)	0 (0.0)	0 (0.0)
Education, n (%)																		
No schooling or primary incomplete	35 (16.1)	15 (31.9)	20 (11.7)	16 (16.2)	13 (18.6)	3 (10.3)	75 (26.3)	55 (33.1)	20 (16.8)	51 (23.6)	37 (26.1)	14 (18.9)	25 (20.5)	8 (44.4)	17 (16.4)	16 (19.8)	13 (23.2)	3 (12.0)
Primary complete or secondary incomplete	118 (54.1)	25 (53.2)	93 (54.4)	33 (33.3)	19 (27.1)	14 (48.3)	161 (56.5)	88 (53.0)	73 (61.3)	95 (44.0)	52 (36.6)	43 (58.1)	54 (44.2)	5 (27.8)	49 (47.1)	34 (42.0)	22 (39.3)	12 (48.0)
Secondary incomplete or more	65 (29.8)	7 (14.9)	58 (33.9)	50 (50.5)	38 (54.3)	12 (41.4)	49 (17.2)	23 (13.9)	26 (21.9)	70 (32.4)	53 (37.3)	17 (23.0)	43 (35.3)	5 (27.8)	38 (36.5)	31 (38.2)	21 (37.5)	10 (40.0)
Employment status, n (%)																		
Employed	57 (26.2)	7 (14.9)	50 (29.2)	34 (34.3)	27 (38.6)	7 (24.1)	69 (24.2)	38 (22.9)	31 (26.1)	67 (31.0)	52 (36.6)	15 (20.3)	26 (21.3)	5 (27.8)	21 (20.2)	26 (32.1)	19 (33.9)	7 (28.0)
Not employed	161 (73.9)	40 (85.1)	121 (70.8)	65 (65.7)	43 (61.4)	22 (75.9)	216 (75.8)	128 (77.1)	88 (73.9)	149 (69.0)	90 (63.4)	59 (79.7)	96 (78.7)	13 (72.2)	83 (79.8)	55 (67.9)	37 (66.1)	18 (72.0)
Expenditure, n (%)																		
1st quintile	28 (12.9)	11 (23.4)	17 (9.9)	17 (17.2)	14 (20.0)	3 (10.3)	75 (26.2)	54 (32.5)	21 (17.5)	48 (22.2)	35 (24.7)	13 (17.6)	21 (17.5)	4 (22.2)	17 (29.8)	14 (17.3)	13 (23.2)	1 (4.0)
2nd quintile	38 (17.5)	15 (31.9)	23 (13.4)	10 (10.1)	4 (5.7)	6 (20.7)	76 (26.6)	52 (31.3)	24 (20.0)	51 (23.6)	28 (19.7)	23 (31.1)	12 (10.0)	5 (27.7)	7 (0.0)	18 (22.2)	11 (19.6)	7 (28.0)
3rd quintile	44 (20.3)	12 (25.5)	32 (18.6)	24 (24.2)	12 (17.1)	12 (41.4)	57 (19.9)	29 (17.5)	28 (23.3)	41 (19.0)	28 (19.7)	13 (17.6)	21 (17.5)	2 (11.1)	19 (0.0)	17 (21.0)	10 (17.9)	7 (28.0)
4th quintile	53 (24.4)	7 (14.9)	46 (26.7)	21 (21.2)	18 (25.7)	3 (10.3)	43 (15.0)	15 (9.0)	28 (23.3)	42 (19.4)	24 (16.9)	18 (24.3)	28 (23.3)	4 (22.2)	24 (24.0)	13 (16.1)	9 (16.1)	4 (16.0)
5th quintile	55 (24.9)	2 (4.3)	53 (30.8)	27 (27.3)	22 (31.4)	5 (17.2)	33 (11.5)	15 (9.0)	18 (15.0)	34 (15.7)	27 (19.0)	7 (9.5)	36 (30.0)	1 (5.6)	35 (41.3)	18 (22.2)	12 (21.4)	6 (24.0)
Missing	1 (0.0)	0 (0.0)	1 (0.6)	0 (0.0)	0 (0.0)	0 (0.0)	2 (0.7)	1 (0.6)	1 (0.8)	0 (0.0)	0 (0.0)	0 (0.0)	2 (1.7)	2 (11.1)	0 (0.0)	1 (1.2)	1 (1.8)	0 (0.0)
Do you belong to a medical aid scheme or any health insurance scheme or programme? n (%)																		
No	211 (96.8)	47 (100)	164 (95.9)	73 (73.7)	51 (72.9)	22 (75.9)	279 (97.9)	163 (98.2)	116 (97.5)	189 (87.5)	120 (84.5)	69 (93.2)	119 (97.5)	17 (94.4)	102 (98.1)	71 (87.7)	49 (87.5)	22 (88.0)
Yes	7 (3.2)	0 (0.0)	7 (4.1)	26 (26.3)	19 (27.1)	7 (24.1)	6 (2.1)	3 (1.8)	3 (2.5)	27 (12.5)	22 (15.5)	5 (6.8)	3 (2.5)	1 (5.6)	2 (1.9)	10 (12.3)	7 (12.5)	3 (12.0)
Does anyone in your family or any close friend have any cancer now or in the past? n (%)																		
No	78 (35.8)	32 (68.1)	46 (26.9)	58 (58.6)	37 (52.9)	21 (72.4)	167 (58.6)	111 (66.9)	56 (47.1)	131 (60.7)	83 (58.5)	48 (64.9)	45 (36.9)	10 (55.6)	35 (33.7)	48 (59.3)	31 (55.4)	17 (68.0)
Yes	133 (61.0)	11 (23.4)	122 (71.4)	37 (37.4)	29 (41.4)	8 (27.6)	97 (33.9)	40 (24.1)	57 (47.9)	71 (32.9)	47 (33.1)	24 (32.4)	68 (55.7)	5 (27.8)	63 (60.6)	20 (24.7)	15 (26.8)	5 (20.0)
Not sure	7 (3.2)	4 (8.5)	3 (1.8)	4 (4.0)	4 (5.7)	0 (0.0)	21 (7.4)	15 (9.0)	6 (5.0)	14 (6.5)	12 (8.4)	2 (2.7)	8 (6.6)	2 (11.1)	6 (5.8)	13 (16.0)	10 (17.9)	3 (12.0)
Missing	0 (0.0)	0 (0.0)	0 (0.0)	0 (0.0)	0 (0.0)	0 (0.0)	1 (0.3)	0 (0.0)	0 (0.0)	0 (0.0)	0 (0.0)	0 (0.0)	1 (0.8)	1 (5.6)	0 (0.0)	0 (0.0)	0 (0.0)	0 (0.0)
History of breast self-examination or cervical screening, n (%)																		
No	91 (41.7)	12 (25.5)	79 (46.2)	41 (41.4)	31 (44.3)	10 (34.5)	21 (6.3)	14 (8.4)	7 (5.8)	45 (20.7)	42 (29.4)	3 (4.1)	NA	NA	NA	NA	NA	NA
Yes	127 (58.3)	35 (74.5)	92 (53.8)	56 (56.6)	38 (54.3)	18 (62.1)	264 (92.6)	152 (91.6)	112 (94.2)	171 (78.8)	100 (69.9)	71 (95.9)	NA	NA	NA	NA	NA	NA
Missing	0 (0.0)	0 (0.0)	0 (0.0)	2 (2.0)	1 (1.4)	1 (3.5)	0 (0.0)	0 (0.0)	0 (0.0)	0 (0.0)	0 (0.0)	0 (0.0)	NA	NA	NA	NA	NA	NA
Self-reported history of clinical breast examination, n (%)																		
No	29 (13.3)	4 (8.5)	25 (14.6)	42 (42.2)	42 (60.0)	0 (0.0)	NA	NA	NA	NA	NA	NA	NA	NA	NA	NA	NA	NA
Yes	189 (86.7)	43 (91.5)	146 (85.4)	56 (56.6)	28 (40.0)	28 (96.6)	NA	NA	NA	NA	NA	NA	NA	NA	NA	NA	NA	NA
Missing	0 (0.0)	0 (0.0)	0 (0.0)	1 (1.0)	0 (0)	1 (3.5)	NA	NA	NA	NA	NA	NA	NA	NA	NA	NA	NA	NA

NA, not available.

### Symptom and risk factor awareness and barriers to seeking care

Online supplemental file 1 provides information on symptom and risk factor awareness, barriers to seeking care and interpretation and emotional response to initial symptoms. For all cancers, symptom and risk factor recall was low with higher proportions of SA compared with Zimbabwean participants unable to recall any symptoms or risk factors.

Across the cancers, participants in Zimbabwe versus SA, and less-resourced versus better-resourced regions reported more barriers to seeking care. A high proportion of Zimbabwean participants reported financial barriers to seeking care, with those living in Bulawayo reporting greater financial barriers compared with participants in Harare (breast 75.9% vs 38.6%, cervical 75.7% vs 50.0%, colorectal 80.0% vs 62.5%). In SA, financial barriers were more frequently reported in the resource-limited EC than WC (cervical cancer 33.1% vs 34.5%, breast cancer 40.4% vs 19.9% and CRC 33.3% vs 16.4%). For all three cancers, more participants living in EC compared with WC reported the distance to the clinic/health facility as a barrier to seeking care (breast 34.0% vs 11.1%, cervical 7.8% vs 4.2%, colorectal 16.7% vs 6.7%).

### Initial symptom experience and interpretation

Overall, 88.0% of breast, 77.2% cervical and 81.7% of CRC patients sought care because of symptoms. A breast lump was the most common first symptom noticed for those diagnosed with breast cancer in both countries (Zimbabwe 75.8%, SA 74.5%). For Zimbabwean participants with cervical cancer, the most reported symptom was persistent smelly vaginal discharge (28.0%), whereas in SA, vaginal bleeding was most reported (34.1%). For both countries, persistent abdominal pain was the most reported first symptom for CRC (Zimbabwe 26.4%, SA 27.7%). The degree to which interpretation of a symptom as serious differed across cancers and regions (online supplemental file 1). In both countries, cervical symptoms were interpreted as most serious, with breast symptoms interpreted as least serious in Zimbabwe and colorectal symptoms least serious in SA.

### Time to diagnosis

Participants were better able to provide an exact date for the first healthcare visit compared with the date a symptom was first noticed. For the date a symptom was first noticed, approximate timeframes (rather than exact dates) were provided for 71.4%, 22.6% and 62.5% of breast, cervical and CRC Zimbabwean participants, respectively, and for 39.6%, 65.3% and 64.8% of SA participants, respectively. For the date of first health provider visit, approximate timeframes were provided for 12.4%, 3.3% and 18.8% of breast, cervical and CRC Zimbabwean participants, respectively, and for 22.0%, 48.3% and 57.9% of SA participants, respectively. The exact date of diagnosis was known for all Zimbabwean and 97.4% of SA participants.

**Figure 1:**
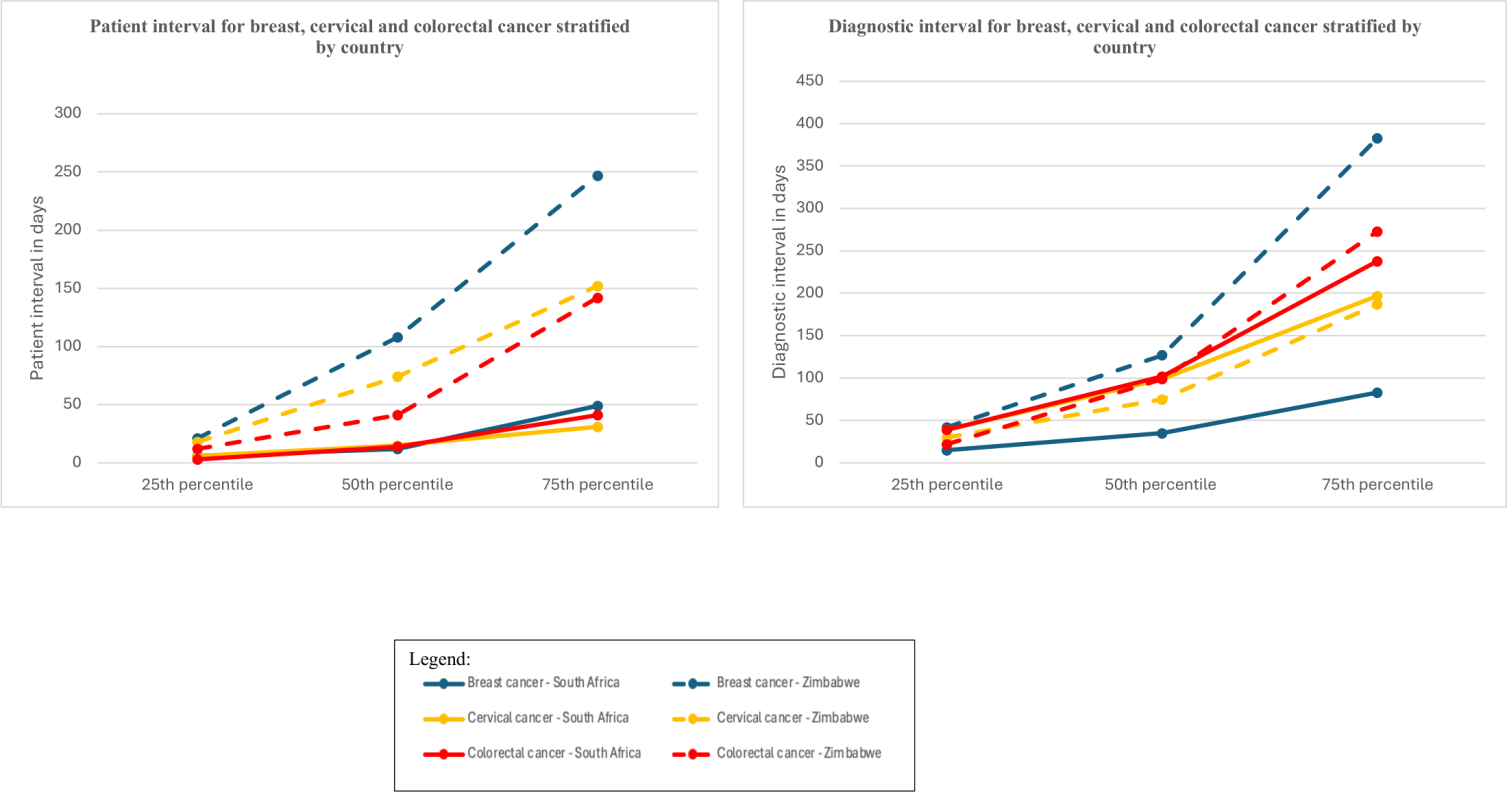
Patient interval and diagnostic interval for breast, cervical and colorectal cancer stratified by country.

Diagnosis was based on histology for most participants (Zimbabwe: breast 97.0%, cervix 98.6%, colorectal 80.2%; SA: breast 99.1%, cervix 96.2%, colorectal 91.0%). PIs were only calculated for those that sought care because of symptoms. Data on the PI and DI, health visits and provider seen are provided in table 2 and figure 1. In Zimbabwe, for all cancers, participants reported a median of four healthcare visits before receiving a diagnosis, with a quarter reporting six or more visits for breast and CRC and five or more visits for cervical cancer. In SA, fewer visits were made to obtain a breast cancer diagnosis compared with receiving a cervical or CRC diagnosis (median 2 for breast cancer vs median 4 for both cervical and CRC). The type of first provider seen differed by country and cancer type. In both countries, median DIs were longer than median PIs for all cancers.

**Table 2:** Intervals in days, healthcare visits and first provider seen

Cancer type	Breast						Cervix						Colorectal					
Country	South Africa			Zimbabwe			South Africa			Zimbabwe			South Africa			Zimbabwe		
Country/region	South Africa N=218	Eastern Cape N=47	Western Cape N=171	Zimbabwe N=99	Harare N=70	Bulawayo N=29	South Africa N=285	Eastern Cape N=166	Western Cape N=119	Zimbabwe N=216	Harare N=142	Bulawayo N=74	South Africa N=122	Eastern Cape N=18	Western Cape N=104	Zimbabwe N=81	Harare N=56	Bulawayo N=25
First noticed symptoms to first visit (not spiritual healer)																		
Total, n	172	34	138	72	52	20	208	136	72	155	97	58	83	11	72	66	45	21
Median days (IQR)	12 (6–49)	15 (3–75)	11 (6–47)	108 (21–247)	113 (21-266)	42 (26–229)	15 (6–31)	15 (4–17)	20 (8–63)	74 (18–152)	81 (29–162)	37 (10–117)	14 (3–41)	13.5 (1–15)	14 (4–53)	41 (12–142)	31 (7–130)	45 (26–180)
First visit to diagnosis																		
Total, n	209	44	165	97	69	28	263	153	110	214	140	74	107	13	94	80	56	24
Median days (IQR)	35 (15–83)	104 (50–181)	28 (13–56)	127 (42–383)	89 (30–264)	166 (104–460)	99 (39–197)	120 (59–247)	70 (20–149)	75 (30–187)	62 (30–218)	83 (28–174)	102 (39–238)	163 (60–295)	102 (39–229)	99 (22–273)	63 (21–197)	156 (57–348)
First provider seen (n %)																		
Local clinic nurse	67 (30.7)	34 (72.3)	33 (19.3)	31 (31.3)	21 (30.0)	10 (34.5)	161 (56.5)	127 (76.5)	34 (28.6)	103 (47.7)	63 (44.4)	40 (54.1)	11 (9.2)	5 (27.8)	6 (5.8)	25 (30.9)	15 (26.8)	10 (40.0)
Local clinic doctor	51 (23.4)	7 (14.9)	44 (25.7)	7 (7.1)	6 (8.6)	1 (3.5)	37 (13.0)	5 (3.0)	32 (26.9)	21 (9.7)	15 (10.6)	6 (8.1)	27 (22.5)	2 (11.1)	25 (24.0)	6 (7.4)	5 (8.9)	1 (4.0)
Local hospital doctor	36 (16.5)	3 (6.4)	33 (19.3)	25 (25.3)	14 (20.0)	11 (37.9)	35 (12.3)	15 (9.0)	20 (16.8)	62 (28.7)	39 (27.5)	23 (31.1)	24 (19.7)	5 (27.8)	19 (18.3)	25 (30.9)	16 (28.6)	9 (36.0)
General practitioner	53 (24.3)	3 (6.4)	51 (29.2)	13 (13.1)	7 (10.0)	6 (20.7)	42 (14.7)	15 (9.0)	27 (22.7)	8 (3.7)	4 (2.8)	4 (5.4)	50 (41.0)	3 (16.7)	47 (45.2)	9 (11.1)	7 (12.5)	2 (8.0)
Specialist at referral hospital	7 (3.2)	0 (0.0)	7 (4.1)	3 (3.0)	3 (4.3)	0 (0.0)	1 (0.4)	0 (0.0)	1 (0.8)	3 (1.4)	3 (2.1)	0 (0.0)	4 (3.3)	0 (0.0)	4 (3.9)	4 (4.9)	4 (7.1)	0 (0.0)
Spiritual healer	0 (0.0)	0 (0.0)	0 (0.0)	11 (11.1)	11 (15.7)	0 (0.0)	1 (0.4)	0 (0.0)	1 (0.8)	12 (5.6)	12 (8.5)	0 (0.0)	2 (1.6)	2 (11.1)	0 (0.0)	10 (12.4)	8 (14.3)	2 (11.1)
Other	4 (1.8)	0 (0.0)	4 (2.3)	8 (8.1)	7 (10.0)	1 (3.5)	8 (2.8)	4 (2.4)	4 (3.4)	6 (2.8)	6 (4.2)	0 (0.0)	2 (1.6)	0 (0.0)	2 (1.9)	1 (1.2)	0 (0.0)	1 (4.0)
Unknown/ missing	0 (0.0)	0 (0.0)	0 (0.0)	1 (1.0)	1 (1.4)	0 (0.0)	0 (0.0)	0 (0.0)	0 (0.0)	1 (0.5)	0 (0.0)	1 (1.4)	2 (1.6)	1 (5.6)	1 (1.0)	1 (1.2)	1 (1.8)	0 (0.0)

The median PIs for all three cancers were shorter in SA than in Zimbabwe (figure 1). In Zimbabwe, the longest PI was for breast cancer (median 108, IQR 21–247). Although the PI among Zimbabwean participants was shorter for cervical cancer (median 74, IQR 18–152) and CRC (median 41, IQR 12–142), a quarter of participants took longer than 5.0 months and 4.7 months, respectively, to visit a first healthcare provider for symptoms.

For breast cancer, the median DI in SA was shorter than in Zimbabwe, while for colorectal and cervical cancer, the median DIs were longer. For breast cancer overall, 42.2% of participants had a DI of ≥60 days: Zimbabwe 60.8%, SA 33.5%. More participants in less-resourced regions experienced DIs of ≥60 days for breast cancer compared with those in better-resourced regions: EC versus WC (72.7% vs 23.0%) and Bulawayo versus Harare (78.6% vs 53.6%).

Factors associated with the patient and DIs on univariate analysis are shown in table 3. Participants living in SA compared with Zimbabwe had shorter PIs for all three cancers and a shorter breast cancer DI. In SA, those living in the WC compared with EC had a longer cervical cancer PI but a shorter DI for breast and cervical cancer. Participants living in Harare compared with Bulawayo had a longer cervical cancer PI and a shorter CRC DI. Greater emotional response and interpretation of symptoms were associated with shorter PIs for breast and cervical cancer, while symptom recall and total number of barriers reported was associated with longer PIs for all three cancers. HIV positive participants had a longer breast DI and shorter colorectal DI. For all three cancers, a higher number of healthcare visits prior to diagnosis was associated with longer DIs. Seeing a doctor or more specialist first provider compared with a local clinic nurse was associated with shorter DIs for all three cancers.

**Table 3:** Factors associated with patient and diagnostic interval on univariate analysis

Variable	Level	Breast		Cervical		Colorectal	
		PI HR (95% CI)	DI HR (95% CI)	PI HR (95% CI)	DI HR (95% CI)	PI HR (95% CI)	DI HR (95% CI)
Country	Zimbabwe versus South Africa	0.44 (0.33 to 0.58)***	0.46 (0.36 to 0.6)***	0.48 (0.39 to 0.6)***	1.12 (0.94 to 1.35)	0.61 (0.44 to 0.85)**	1.09 (0.82 to 1.46)
Province	Western Cape versus Eastern Cape	1.27 (0.86 to 1.88)	2.57 (1.81 to 3.64)***	0.68 (0.51 to 0.9)**	1.51 (1.18 to 1.94)**	0.7 (0.37 to 1.34)	1.36 (0.76 to 2.46)
	Harare versus Bulawayo	1.02 (0.6 to 1.73)	1.16 (0.74 to 1.81)	0.79 (0.57 to 1.09)°	0.94 (0.71 to 1.25)	1.37 (0.81 to 2.33)	1.72 (1.05 to 2.82)*
Age	–	1.01 (1 to 1.02)°	1.00 (0.99 to 1.01)	1.00 (0.99 to 1.01)	1.01 (1 to 1.01)°	1.01 (1 to 1.02)*	1.00 (1 to 1.01)
Sex	Male	–	–	–	–	0.97 (0.7 to 1.34)	1.15 (0.86 to 1.54)
Household size	–	0.96 (0.9 to 1.02)°	0.99 (0.94 to 1.05)	1.00 (0.96 to 1.05)	1.00 (0.98 to 1.02)	0.98 (0.91 to 1.06)	0.99 (0.93 to 1.06)
Expenditure index	2	1.04 (0.64 to 1.69)	0.83 (0.55 to 1.26)	0.94 (0.7 to 1.26)	1.02 (0.79 to 1.32)	1.13 (0.63 to 2)	0.66 (0.39 to 1.1)
	3	0.97 (0.63 to 1.51)	0.79 (0.54 to 1.16)	0.77 (0.56 to 1.05)	1.03 (0.78 to 1.35)	1.43 (0.81 to 2.52)	0.93 (0.56 to 1.52)
	4	1.01 (0.66 to 1.56)	0.94 (0.64 to 1.37)	0.89 (0.64 to 1.23)	1.28 (0.97 to 1.7)	1.21 (0.69 to 2.1)	0.93 (0.58 to 1.5)
	5	1.05 (0.69 to 1.61)	1.14 (0.79 to 1.64)	1.01 (0.71 to 1.43)	1.14 (0.84 to 1.55)	1.19 (0.71 to 1.99)	1.10 (0.7 to 1.73)
Relationship status	Separated, divorced or widowed	0.91 (0.68 to 1.21)’	0.84 (0.65 to 1.08)	0.83 (0.65 to 1.05)*	1.16 (0.94 to 1.43)	0.88 (0.6 to 1.29)	0.8 (0.58 to 1.11)*
	Single	1.41 (1 to 1.98)’	1.08 (0.79 to 1.46)	1.25 (0.95 to 1.64)*	1.14 (0.9 to 1.44)	0.93 (0.58 to 1.49)	1.46 (0.95 to 2.26)*
Educational status	No schooling or primary incomplete	1.08 (0.72 to 1.62)	0.87 (0.62 to 1.23)	1.11 (0.83 to 1.5)*	1.00 (0.77 to 1.29)	0.86 (0.53 to 1.39)*	0.75 (0.49 to 1.14)
	Primary complete or secondary incomplete	1.06 (0.81 to 1.4)	1.14 (0.89 to 1.46)	1.37 (1.06 to 1.78)*	0.92 (0.74 to 1.15)	1.44 (0.99 to 2.08)*	1.08 (0.78 to 1.48)
Employment status	Not employed	1.09 (0.83 to 1.43)	0.91 (0.71 to 1.16)	1.13 (0.88 to 1.44)	0.93 (0.76 to 1.14)	1 (0.69 to 1.45)	0.76 (0.54 to 1.05)’
Medical insurance	Yes	0.88 (0.58 to 1.33)	0.71 (0.49 to 1.01)’	0.93 (0.59 to 1.47)	1.22 (0.84 to 1.76)	1.37 (0.69 to 2.7)	1.76 (1 to 3.12)’
Regular self-breast check	Yes	1.37 (1.01 to 1.86)*	1.09 (0.83 to 1.43)	–	–	–	–
Cancer in family or friends	No	0.69 (0.53 to 0.9)**	0.65 (0.52 to 0.81)***	0.89 (0.71 to 1.11)	0.84 (0.7 to 1.02)’	0.81 (0.58 to 1.13)	0.84 (0.63 to 1.13)
HIV status	Unknown	1.01 (0.7 to 1.46)	0.85 (0.62 to 1.17)**	0.83 (0.53 to 1.28)	1.09 (0.75 to 1.59)	1.34 (0.91 to 1.97)	1.27 (0.9 to 1.78)°
	Positive	0.92 (0.66 to 1.27)	0.61 (0.45 to 0.83)**	0.95 (0.77 to 1.18)	0.98 (0.82 to 1.19)	1.12 (0.66 to 1.91)	1.44 (0.88 to 2.33)°
First symptom noticed	Bleeding	–	–	–	–	0.96 (0.63 to 1.47)	0.67 (0.44 to 1.01)°
	Bowel habits	–	–	–	–	1.34 (0.8 to 2.26)	0.63 (0.39 to 1.03)°
	Discharge	–	–	0.88 (0.64 to 1.23)	1.12 (0.81 to 1.54)’	–	–
	Lump	0.92 (0.61 to 1.38)	1.12 (0.74 to 1.68)	–	–	–	–
	Vaginal bleeding	–	–	0.85 (0.64 to 1.14)	1.43 (1.07 to 1.9)’	–	–
	Other	0.88 (0.51 to 1.5)	1.10 (0.64 to 1.88)	0.74 (0.48 to 1.15)	1.26 (0.83 to 1.92)’	1.14 (0.73 to 1.76)	0.83 (0.54 to 1.27)°
Emotional response score	–	1.12 (1.08 to 1.17)***	–	1.07 (1.03 to 1.12)***	–	0.97 (0.92 to 1.02)	–
Symptom interpretation score	–	1.14 (1.09 to 1.2)***	–	1.08 (1.03 to 1.12)***	–	1.01 (0.96 to 1.07)	–
Total barriers	–	0.9 (0.85 to 0.96)**	0.93 (0.88 to 0.98)**	0.91 (0.86 to 0.96)***	1.00 (0.96 to 1.05)	0.94 (0.88 to 1.01)’	0.98 (0.93 to 1.04)
Risk factor recall	–	0.96 (0.72 to 1.28)	0.96 (0.74 to 1.26)	0.75 (0.6 to 0.94)*	0.99 (0.84 to 1.17)	0.83 (0.63 to 1.09)°	1.25 (0.99 to 1.59)’
Symptom recall	–	0.85 (0.76 to 0.95)**	0.91 (0.82 to 1)’	0.81 (0.71 to 0.91)***	1.03 (0.94 to 1.13)	0.77 (0.66 to 0.91)**	1.03 (0.89 to 1.19)
First provider seen	General practitioner	–	1.31 (0.95 to 1.8)’	–	1.26 (0.92 to 1.72)*	–	1.11 (0.72 to 1.71)*
	Local clinic doctor	–	1.59 (1.14 to 2.21)’	–	1.42 (1.06 to 1.91)*	–	1.34 (0.82 to 2.18)*
	Local hospital doctor	–	1.32 (0.95 to 1.82)’	–	0.99 (0.78 to 1.26)*	–	1.25 (0.8 to 1.96)*
	Specialist at referral hospital	–	1.62 (0.75 to 3.5)’	–	2.86 (0.91 to 8.97)*	–	4.05 (1.84 to 8.9)*
	Spiritual healer	–	1.07 (0.57 to 2.01)’	–	0.91 (0.52 to 1.6)*	–	0.83 (0.41 to 1.69)*
	Unknown	–	0.74 (0.4 to 1.35)’	–	0.65 (0.36 to 1.17)*	–	1.27 (0.39 to 4.15)*
Number of visits prior to diagnosis	–	–	0.77 (0.71 to 0.82)***	–	0.77 (0.73 to 0.81)***	–	0.84 (0.79 to 0.89)***
Complete cases		239	304	361	372	148	156

P value from Wald test is in a range 0.1–0.2 if °, 0.05–0.1 if ', 0.01–0.05 if *, 0.001–0.01 if **, <0.001 if ***.

DI, diagnostic interval; PI, patient interval.

Online supplemental file 2a–e provides information on the multivariable analysis of factors associated with the PI. Experiencing more barriers to seeking care was associated with longer breast cancer PIs in both countries (Zimbabwe HR 0.801, 95% CI 0.703 to 0.913; SA HR 0.885, 95% CI 0.817 to 0.958), and also with a longer cervical cancer PI in Zimbabwe (HR 0.901, 95% CI 0.828 to 0.979). In SA, WC participants had a longer PI for cervical cancer compared with the EC (HR 0.677, 95% CI 0.507 to 0.905). Greater emotional response to a symptom was associated with shorter PIs for breast cancer (Zimbabwe HR 1.194, 95% CI 1.101 to 1.295; SA HR 1.145, 95% CI 1.079 to 1.216). Shorter PIs were also associated with interpreting a cervical symptom as more serious in Zimbabwe (HR 1.130, 95% CI 1.057 to 1.209). In Zimbabwe, being single compared with being married/living in a partnership was associated with a shorter breast cancer PI (HR 4.413, 95% CI 1.688 to 11.539). Factors associated with a longer PI for CRC included living in Zimbabwe versus SA (HR 0.675, 95% CI 0.478 to 0.952) and symptom recall (HR 0.776, 95% CI 0.653 to 0.923).

The Cox regression analysis indicated that a higher number of healthcare visits prior to diagnosis was significantly associated with longer DIs for all cancers in both countries (online supplemental file 3a–f). For breast cancer in SA, those with unknown compared with HIV negative status had a longer DI (HR 0.577, 95%CI 0.407 to 1.020). In SA, living in the WC versus the EC was associated with a shorter DI for breast (HR 2.458, 95% CI 1.651 to 3.660) and cervical cancer (HR 1.657, 95% CI 1.172 to 2.344), but not for CRC. The type of provider first seen impacted the DI. In SA, shorter DIs were associated with seeing a clinic doctor versus a nurse for breast cancer (HR 1.524, 95% CI 1.030 to 2.255) and seeing a referral hospital specialist provider versus a local clinic nurse for CRC (HR 4.071, 95% CI 1.141 to 14.523). In Zimbabwe, first seeing a local clinical doctor versus seeing the local clinic nurse had a shorter CRC DI (HR 3.040, 95% CI 1.077 to 8.583) but a longer breast cancer DI (HR 0.250, 95% CI 0.096 to 0.648).

## Discussion

In the Southern African context, where advanced stage at diagnosis and poor outcomes are common, improving timely diagnosis is crucial. To achieve this, a clear understanding of the diagnostic timeline is essential. This study provides important insights into the time to diagnosis and associated factors among patients with breast, cervical and CRC in Zimbabwe and SA.

We found that symptom and risk factor awareness, important in initiating and navigating the journey to cancer diagnosis, was low for all three cancers, particularly among SA participants. This is concerning given our study population of people diagnosed with cancer. Consistent with other studies set in Africa, we found that initially interpreting a symptom as more serious and being more concerned about initial symptoms was associated with a shorter PI.^19 20^ Our findings, together with results from other studies in Africa, highlight the urgent need for targeted health interventions that improve symptom recognition while guiding individuals towards appropriate help-seeking behaviour.^21^,^24^ Awareness interventions such as health education and awareness promotion campaigns could be effective in improving public knowledge and help-seeking behaviour in Southern Africa and other LMICs.^25^ Evidence from the UK further supports early diagnosis interventions targeting common cancer symptoms (eg, breast or axillary lump for breast cancer, abnormal vaginal bleeding for cervical cancer and rectal bleeding for CRC) to improve cancer outcomes.^26^

The PI varied across cancer types and geographical regions, with Zimbabwean participants experiencing longer PIs than SA participants. The longest PI in our study was 108 days for breast cancer patients in Zimbabwe. This is much longer than has been reported for most breast cancer studies in Africa.^20 27 28^ For breast and cervical cancer, a systematic review found pooled median PIs of 58 and 79 days for breast and cervical cancer in lower-income countries,^29^ while a study in Mexico City reported median PIs of 19 and 24 days for breast and cervical cancer.^30^ Given the more visible presentation of breast cancer symptoms compared with cervical and CRC, the long PI for breast cancer in Zimbabwe is particularly concerning. Petrova *et al* reported a pooled median PI of 90 days for CRC patients in lower-income countries.^29^ Our study reported much shorter median PIs (SA 14 days, Zimbabwe 41 days); however, a quarter of the Zimbabwean CRC participants had a worrying PI exceeding 142 days.

For all three cancers, participants in less-resourced regions reported more barriers to seeking care compared with those in better-resourced areas and DIs were longer in less-resourced regions, reflecting disparities in healthcare infrastructure, transport and other inequities that impact cancer outcomes.^31 32^ Similar to others, we found that financial constraints were a key barrier,^22 30 33 34^ particularly for those living in Zimbabwe and in the EC. Barriers to seeking care significantly impacted PIs for breast cancer in both countries and cervical cancer in Zimbabwe, pointing to the urgent need to address financial and other barriers to seeking care.

A strength of our study is the use of a methodological framework, validated tools and widely supported methods for reporting on cancer timelines in addition to a multisite and multicountry approach. Fewer LMIC studies on time to cancer diagnosis measure DIs compared with PIs, possibly reflecting the biased view that delays in diagnosis are mainly due to patient factors.^29 35^ An additional strength of our study was that both patient and health system-related intervals were measured. In both countries and for all three cancers, we found that the DIs were much longer than PIs, indicating that substantial delays occur after the first healthcare visit. In our study, the median DIs all exceeded 1 month: for breast, cervical and CRC in Zimbabwe, median DIs were 127, 75 and 99 days, respectively, and in SA 35, 99 and 102 days, respectively. By comparison, Petrova *et al*^29^ reported median DIs of 55, 97 and 67 days for breast, cervical and CRC respectively in lower-income countries. The African Breast Cancer-Disparities in Outcomes (ABC-DO) study, which examined breast cancer pathways in several African countries, reported median DIs ranging from 0.3 to 3.7 months (Namibia 0.3 and 1.3 months, Zambia 1.1 months, Uganda 3.5 months and Nigeria 3.7 months).^36^ The SA participants in our study had DIs comparable to Namibia and Zambia, whereas Zimbabwean participants had much longer DIs than reported by any of the ABC-DO study countries.

In 2023, the WHO launched the Global Breast Cancer Initiative (GBCI) Framework which aims to reduce breast cancer mortality by 2.5% annually and prevent 2.5 million breast cancer deaths globally by 2040.^37^ One of the GBCI key performance indicators (KPIs) targets a DI of under 60 days. However, data on this KPI are scarce: most countries in LMICs do not routinely measure or capture such data in cancer registries and studies reporting on breast cancer DI usually report on the median. We found that 67% of SA and 39% of Zimbabwean participants met this GBCI target. In comparison, the ABC-DO study reported between 20% and 66% of countries reached this target.^36^ Given that longer DIs are associated with worse cancer outcomes, urgent interventions that target this KPI are needed in the region. Studies of interventions aimed at reducing the DI in LMICs suggest that improving provider clinical and diagnostic knowledge and competencies, patient navigation programmes and the introduction of one-stop clinics may be feasible and effective in LMIC settings.^25^

Interventions to shorten the DI must consider the health system context, particularly for those living in rural areas. Most participants in our study visited an average of four healthcare providers before receiving a diagnosis, with a higher number of visits associated with longer DIs. Understanding the actions and decisions at each healthcare visit and the interval between each visit is important to reducing the DI.^38^ The type of provider first seen influenced the DIs, with shorter intervals reported where the first provider was a clinic doctor or a referral level specialist compared with a clinic nurse. This is of relevance, given that for most patients in the African region, the first healthcare provider seen is a nurse, with doctors making infrequent visits to primary care clinics. Our findings support the call to better support front line healthcare providers with interventions that support symptom assessment and management and improve referral pathways.^27 34 35^

Our hospital-based study may have introduced selection bias by excluding individuals who did not reach the recruiting hospitals within the 1 month period after diagnosis. As a result, our findings do not fully reflect the experiences of all cancer patients, particularly those facing significant barriers to diagnosis. We relied on patients self-reported date of first noticing a symptom and first healthcare visit, which could have been affected by recall bias. To minimise recall bias, we interviewed patients within 1 month of diagnosis and used calendar prompts to aid patients’ memory. Efforts to measure time to diagnosis intervals are growing in LMICs. They rely on accurate measures of the beginning and end points of each interval. While the Aarhus statement has provided guidance on standardised approaches, there are challenges in implementing the guidelines.^7 39^ We found that in our setting, even with calendar landmarking techniques, recall of exact dates for onset of symptoms and date of first health provider visit was challenging. To our knowledge, there is no guidance in the literature on how best to estimate intervals using approximate timeframes rather than exact dates. We developed a method to handle this data, but this will need to be tested in future studies.

In conclusion, our study highlights the urgent need to improve timely cancer diagnosis in Southern Africa, where both patient and health system delays contribute to diagnostic timelines. Efforts should focus on enhancing cancer symptom awareness and interpretation, addressing barriers to accessing care and supporting primary healthcare providers in managing and referring symptomatic patients.

## Supplementary material

10.1136/bmjgh-2025-021889 online supplemental file 1

10.1136/bmjgh-2025-021889 online supplemental file 2

## Data Availability

Data are available on reasonable request.
