# Supplementary material for: Time to diagnosis for breast, cervical and colorectal cancer in Zimbabwe and South Africa: a cross-sectional study
Source: BMJ Glob Health. 2026 Feb 11;11(2):e021889. doi: 10.1136/bmjgh-2025-021889 (PMC12911819; doi:10.1136/bmjgh-2025-021889)
Supplement: online supplemental file 1 [file bmjgh-11-2-s001.pdf]

REFLEXIVITY STATEMENT

1. How does this study address local research and policy priorities?

Breast, cervical and colorectal cancers are important causes of morbidity and mortality in Southern Africa. International evidence shows that shorter time to cancer diagnosis is associated with better outcomes, yet little is known about these intervals in Southern Africa. Understanding and accurately measuring the time interval from symptom discovery to cancer diagnosis is important in guiding development and evaluation of early diagnosis interventions This study determined the time from breast, cervical and colorectal cancer symptom awareness to presentation in healthcare and diagnosis, and factors influencing these intervals in two Southern African countries, Zimbabwe and South Africa (SA). Results are being used to develop and evaluated early diagnosis interventions.

2. How were local researchers involved in study design?

This study was conceptualized by JM (South Africa) and FMW (United Kingdom) and builds on an earlier collaborative 3-year project. The study was designed with inputs from the research team based in South Africa, Zimbabwe and the UK. Our large team of local collaborators also contributed to the study design, providing comments on the study instruments and data recruitment methods and supporting data collection.

3. How has funding been used to support the local research team?

The AWACAN-ED program is funded by a NIHR Global Health award (NIHR133231, £2,991,774.00), with most of the funding allocated to the South African and Zimbabwean teams. Funding has been used to recruit and train local field staff, collect data, provide student bursaries (see below), host an annual one-week research school (see below), and to support team members to present findings at local and international conferences.

4. How are research staff who conducted data collection acknowledged?

Local staff who oversaw the data collection are included as co-authors (SD, BTG). The field teams are recognised in the acknowledgement statement.

5. Do all members of the research partnership have access to study data?

All members of the partnership have access to the data.

6. How was data used to develop analytical skills within the partnership?

Capacity development is a key aim of this partnership. Results were presented and discussed with all team members at fortnightly online meetings. Senior investigators mentor and co-supervise all research staff and students working with data from this study. Training on data analysis was included in the Southern African Cancer Research School (see below).

7. How have research partners collaborated in interpreting study data?

Results were presented, discussed and interpreted with research team members at fortnightly meetings. A series of workshops were held to discuss findings with the larger collaborating team. Results were also presented to our Steering Committee and External Advisory Board regular meetings. Inputs from all these interactions contributed to the interpretation of our results.

8. How were research partners supported to develop writing skills?

Senior academic staff worked with the research partners to develop and refine their writing skills. Sessions on academic research writing were included in the Southern African Cancer Research School (see below).

9. How will research products be shared to address local needs?

Results of the have been discussed with local collaborators and policy makers in South Africa and Zimbabwe. Interventions to improve public awareness and support primary care providers were developed based on the results. These interventions are currently being evaluated. Post-evaluation results will be presented to relevant stakeholders in a series of local dissemination workshops.

10. How is the leadership, contribution and ownership of this work by LMIC researchers recognised within the authorship?

Authors JM and FMW worked as part of the senior authorship team in developing this manuscript, and their contribution has been recognised as first and last authors respectively. Our manuscript includes researchers based in Zimbabwe, South Africa and the United Kingdom. Half of our authors are based in Southern Africa and half in the United Kingdom.

11. **How have early career researchers across the partnership been included within the authorship team?**

We have included early career researchers (SD, BTG, EP, SG) within the authorship team. They attended relevant meetings, and contributed to the literature review, data collection, analysis, interpretation and write-up.

12. How has gender balance been addressed within the authorship?

Seven authors are female (JM, SES, SD, DP, EP, VAS, FMW) and five are male (BTG, ZVM, JEA, JMy, SG)

13. How has the project contributed to training of LMIC researchers?

Cross-country training is built into the AWACAN-ED project through:

- Bursaries and mentorship for students. We have three Masters in Public Health and a PhD in Public Health student in South Africa and five Masters and one PhD student in Zimbabwe.
- An annual one-week Southern African School for Cancer Research that supports emerging researchers from the Southern African region. The School includes interactive sessions on research methods, health systems and implementation research, writing and presentation skills as well discussions on career development.
- The AWACAN-ED program includes post-doctoral fellows and emerging researchers as part of the research team. They receive ongoing mentorship.
- Local field teams received on-site training on quantitative and qualitative data collection.

14. How has the project contributed to improvements in local infrastructure?

This project has not directly contributed to improvements in local infrastructure.

15. What safeguarding procedures were used to protect local study participants and researchers?

Approvals were obtained from all relevant ethics and institutional authorities in South Africa and Zimbabwe with due attention to anonymity, confidentiality, non-maleficence, justice, autonomy and respect for persons. Safeguarding referral procedures were put in place to refer any patients requiring psychological or clinical support.
