# Supplementary material for: Time to diagnosis for breast, cervical and colorectal cancer in Zimbabwe and South Africa: a cross-sectional study
Source: BMJ Glob Health. 2026 Feb 11;11(2):e021889. doi: 10.1136/bmjgh-2025-021889 (PMC12911819; doi:10.1136/bmjgh-2025-021889)
Supplement: online supplemental file 2 [file bmjgh-11-2-s002.pdf]

Supplementary appendices

Table of contents

Supplementary figure 1: Model of Pathways to Treatment	1
Supplementary table S1: Symptom and risk factor awareness and barriers to presentation	2-3
Supplementary table S2a: Cox regression results for patient interval: breast cancer, South Africa	4
Supplementary table S2b: Cox regression results for patient interval: breast cancer, Zimbabwe	5
Supplementary table S2c: Cox regression results for patient interval: cervical cancer, South Africa	6
Supplementary table S2d: Cox regression results for patient interval: cervical cancer, Zimbabwe	7
Supplementary table S2e: Cox regression results for patient interval: colorectal cancer, Zimbabwe and South Africa combined	8
Supplementary table S3a: Cox regression results for diagnostic interval: breast cancer, South Africa	9
Supplementary table S3b: Cox regression results for diagnostic interval: breast cancer, Zimbabwe	10
Supplementary table S3b: Cox regression results for diagnostic interval: cervical cancer, South Africa	11
Supplementary table S3d: Cox regression results for diagnostic interval: cervical cancer, South Africa	12
Supplementary table S3e: Cox regression results for diagnostic interval: colorectal cancer, South Africa	13
Supplementary table S3f: Cox regression results for diagnostic interval: colorectal cancer, South Africa	14

Supplementary Figure 1

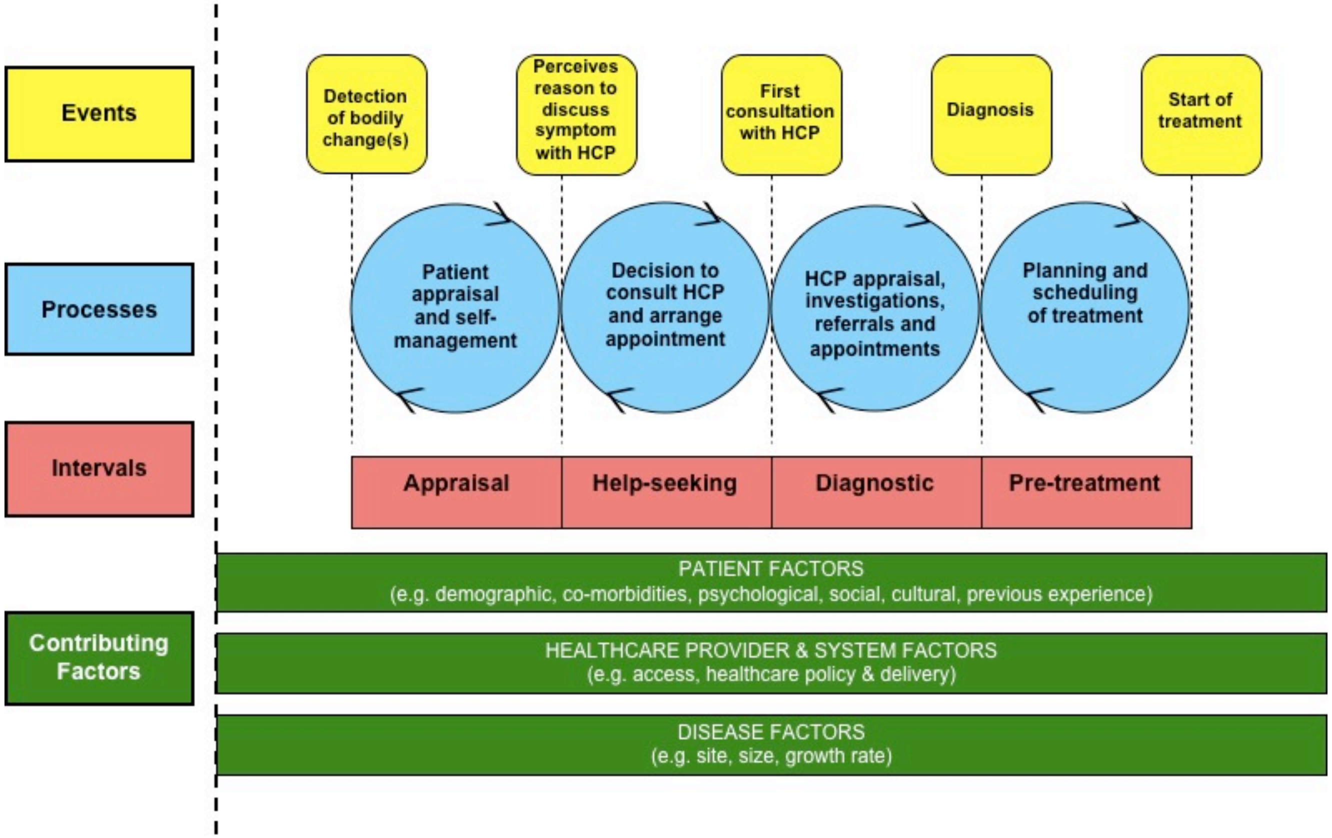

Scott S, Walter F, Webster A, Sutton S, Emery J. The model of pathways to treatment: conceptualization and integration with existing theory. *British Journal of Health Psychology* 2013; **18**(1): 45-65. doi: 10.1111/j.2044-8287.2012.02077.

Walter F, Webster A, Scott S, Emery J. The Andersen Model of Total Patient Delay: a systematic review of its application in cancer diagnosis. *Journal of Health Services Research & Policy* 2012; **17**(2): 110-8. doi: 10.1258/jhsrp.2011.010113.

Supplementary table S1: Symptom and risk factor awareness and barriers to presentation																		
**Cancer type**	**Breast**						**Cervix**						**Colorectal**					
**Country**	**South Africa**			**Zimbabwe**			**South Africa**			**Zimbabwe**			**South Africa**			**Zimbabwe**		
**Region**	South Africa	Eastern Cape	Western Cape	Zimbabwe	Harare	Bulawayo	South Africa	Eastern Cape	Western Cape	Zimbabwe	Harare	Bulawayo	South Africa	Eastern Cape	Western Cape	Zimbabwe	Harare	Bulawayo
Number of symptoms recalled (mean and SD)	0·8 (1·2)	0·3 (0·6)	0·9 (1·3)	1·7 (1·2)	2·0 (1·2)	1·1 (0·8)	0·3 (0·6)	0·1 (0·5)	0·4 (0·7)	1·3 (0·9)	1·5 (0·9)	1·0 (0·8)	0·7 (1·0)	0·3 (0·6)	0·7 (1·0)	1·2 (1·0)	1·1 (1·1)	1·6 (0·6)
Did not recall any symptoms (n %)	130 (59·6)	37 (78·7)	93 (54·4)	10 (10·3)	6 (8·6)	4 (14·8)	230 (80·7)	150 (90·4)	80 (67·2)	42 (19·4)	22 (15·5)	20 (27·0)	68 (55·7)	13 (72·2)	55 (52·9)	12 (14·8)	12 (21·4)	0 (0·0)
Number of risk factors recalled (mean and SD)	0·1 (0·4)	0 (0·1)	0·1 (0·4)	0·2 (0·5)	0·3 (0·5)	0·0 (0·2)	0·1 (0·4)	0·1 (0·3)	0·2 (0·5)	0·4 (0·6)	0·5 (0·6)	0·2 (0·4)	0·2 (0·6)	0·1 (0·2)	0·2 (0·7)	0·3 (0·6)	0·4 (0·7)	0 (0·0)
Did not recall any risk factors (n %)	203 (93·1)	47 (100)	156 (91·2)	80 (82·5)	54 (77·1)	26 (96·3)	260 (91·2)	159 (95·8)	101 (84·9)	148 (68·5)	86 (60·6)	62 (83·8)	102 (83·6)	14 (77·8)	88 (84·6)	52 (64·2)	29 (51·8)	23 (92·0)
Barriers to seeking care (number of barriers agreed with) Mean and SD (out of 16)	1·6 (2·1)	2·4 (2·7)	1·4 (1·8)	2·1 (2·3)	2·4 (2·7)	1·3 (1·1)	1·5 (2·0)	1·5 (1·6)	1·4 (1·9)	2·2 (2·3)	2·5 (2·5)	1·6 (1·6)	1·4 (2·5)	3·1 (3·6)	1·2 (2·1)	2·3 (2·8)	2·7 (3·2)	1·4 (1·4)
Initial interpretation of symptoms (mean and SD) higher value represents more serious initial interpretation	4·9 (2·8)	5·5 (3·1)	4·7 (2·7)	3·3 (2·8)	3·1 (2·8)	3·6 (3·0)	5·0 (2·8)	5·9 (2·5)	3·8 (2·9)	4·6 (2·8)	4·4 (2·6)	4·9 (3·0)	4·2 (2·9)	5·4 (2·6)	4·0 (2·9)	4·2 (2·8)	4·3 (2·7)	4·0 (2·9)
Emotional response to symptoms (mean and SD)	3·1 (2·8)	3·6 (3·3)	2·9 (2·7)	2·6 (3·0)	2·4 (2·8)	3·1 (3·3)	4·0 (3·3)	5·1 (3·0)	2·5 (3·0)	3·9 (3·2)	3·4 (3·0)	5·0 (3·3)	2·7 (3·2)	4·6 (3·7)	2·4 (3·0)	3·9 (2·8)	3·6 (2·8)	4·6 (2·8)

Supplementary table S1: Symptom and risk factor awareness and barriers to presentation

Cancer type	Breast						Cervix						Colorectal					
Country	South Africa			Zimbabwe			South Africa			Zimbabwe			South Africa			Zimbabwe		
higher value represents greater initial emotional response																		

Abbreviations:

SD: Standard deviation

Supplementary table S2a: Cox regression results for patient interval: breast cancer, South Africa		
**Variable**	**HR [95% CI]**	**p-value**
Total barriers	0.885 [0·817, 0·958]	(0·003) **
Emotional Response score	1.145 [1·079, 1·216]	(<0·001) ***
**Model metrics**		
Number of observations	172	
AIC	1413·6	
BIC	1419·9	
RMSE	1·03	

Abbreviations:

HR: hazard ratio

CI: Confidence interval

AIC: Akaike information criterion; BIC: Bayesian information criterion; RMSE: root mean squared error

*** <0.001, ** 0.001-0.01, * 0.01-0.05

Supplementary table 2b: Cox regression results for patient interval: breast cancer, Zimbabwe			
**Variable**	**Categories**	**HR [95% CI]**	**p-value**
Relationship status	single vs married/living with a partner	4·413 [1·688, 11·539]	(0·002) **
	separated/divorced/widowed vs married/living with a partner	0·841 [0·509, 1·390]	(0·499)
Total barriers		0·801 [0·703, 0·913]	(<0·001) ***
Emotional Response score		1·194 [1·101, 1·295]	(<0·001) ***
**Model metrics**			
Number of observations		71	
AIC		449·4	
BIC		458·5	
RMSE		1·01	

Abbreviations:

HR: hazard ratio

CI: Confidence interval

AIC: Akaike information criterion; BIC: Bayesian information criterion; RMSE: root mean squared error

*** <0.001, ** 0.001-0.01, * 0.01-0.05

Supplementary table 2c: **Cox regression results for patient interval: cervical cancer, South Africa**			
**Variable**	**Categories**	**HR [95% CI]**	**p-value**
Province	Western Cape vs Eastern Cape	0·677 [0·507, 0·905]	(0·008) **
**Model metrics**			
Number of observations		208	
AIC		1806·4	
BIC		1809·7	
RMSE		1·01	

Abbreviations:

HR: hazard ratio

CI: Confidence interval

AIC: Akaike information criterion; BIC: Bayesian information criterion; RMSE: root mean squared error

*** <0.001, ** 0.001-0.01, * 0.01-0.05

Supplementary table 2d: Cox regression results for patient interval: cervical cancer, Zimbabwe		
**Variable**	**HR [95% CI]**	**p-value**
Total barriers	0·901 [0·828, 0·979]	(0·014) *
Symptom interpretation score	1·130 [1·057, 1·209]	(<0·001) ***
Risk factor recall	0·673 [0·498, 0·909]	(0·010) **
**Model metrics**		
Number of observations	155	
AIC	1237·7	
BIC	1246·8	
RMSE	0·99	

Abbreviations:

HR: hazard ratio

CI: Confidence interval

AIC: Akaike information criterion; BIC: Bayesian information criterion; RMSE: root mean squared error

*** <0.001, ** 0.001-0.01, * 0.01-0.05

Supplementary table 2e: Cox regression results for patient interval: colorectal cancer, Zimbabwe and South Africa combined			
**Variable**	**Categories**	**HR [95% CI]**	**p-value**
Country	Zimbabwe vs South Africa	0·675 [0·478, 0·952]	(0·025) *
Educational status	primary complete / secondary incomplete vs secondary complete or more	1·484 [1·022, 2·154]	(0·038) *
	no schooling / primary incomplete vs secondary complete or more	0·763 [0·466, 1·247]	(0·280)
Symptom recall		0·776 [0·653, 0·923]	(0·004) **
**Model metrics**			
Number of observations		149	
AIC		1184·0	
BIC		1196·0	
RMSE		0·98	

Abbreviations:

HR: hazard ratio

CI: Confidence interval

AIC: Akaike information criterion; BIC: Bayesian information criterion; RMSE: root mean squared error

*** <0.001, ** 0.001-0.01, * 0.01-0.05

Supplementary table 3a: Cox regression results for diagnostic interval: breast cancer, South Africa			
**Variable**	**Categories**	**HR [95% CI]**	**p-value**
Province	Western Cape vs Eastern Cape	2·458 [1·651, 3·660]	(<0·001) ***
HIV status	positive	0·644 [0·407, 1·020]	(0·061)
	unknown	0·577 [0·407, 1·020]	(0·061)
Number of visits prior to diagnosis		0·621 [0·398, 0·836]	(0·003) **
First provider seen	local clinic doctor vs local clinic nurse	1·524 [1·030, 2·255]	(0·035) *
	local hospital doctor vs local clinic nurse	0·850 [0·536, 1·350]	(0·492)
	general practitioner vs local clinic nurse	0·866 [0·574, 1·307]	(0·495)
	specialist at referral hospital vs local clinic nurse	0·623 [0·219, 1·778]	(0·377)
	unknown vs local clinic nurse	0·612 [0·218, 1·778]	(0·352)
**Model metrics**			
Number of observations		209	
AIC		1741·7	
BIC		1765·1	
RMSE		1·16	

Abbreviations:

HR: hazard ratio

CI: Confidence interval

AIC: Akaike information criterion; BIC: Bayesian information criterion; RMSE: root mean squared error

*** <0.001, ** 0.001-0.01, * 0.01-0.05

Supplementary table 3b: Cox regression results for diagnostic interval: breast cancer, Zimbabwe			
**Variable**	**Categories**	**HR [95% CI]**	**p-value**
Total barriers		0·897 [0·818, 0·985]	(0·022) *
Number of visits prior to diagnosis		0·862 [0·790, 0·940]	(<0·001) ***
First provider seen	local clinic doctor vs local clinic nurse	0·250 [0·096, 0·648]	(0·004) **
	local hospital doctor vs local clinic nurse	0·802 [0·463, 1·392]	(0·434)
	specialist at referral hospital vs local clinic nurse	3·520 [0·990, 12·508]	(0·052) +
	spiritual healer vs local clinic nurse	1·444 [0·707, 2·947]	(0·313)
	unknown vs local clinic nurse	0·423 [0·180, 0·991]	(0·048) *
**Model metrics**			
Number of observations·		97	
AIC		690·0	
BIC		710·6	
RMSE		0·98	

Abbreviations:

HR: hazard ratio

CI: Confidence interval

AIC: Akaike information criterion; BIC: Bayesian information criterion; RMSE: root mean squared error

*** <0.001, ** 0.001-0.01, * 0.01-0.05, + 0.05-0.1

Supplementary table 3c: Cox regression results for diagnostic interval: cervical cancer, South Africa			
**Variable**	**Categories**	**HR [95% CI]**	**p-value**
Province	Western Cape vs Eastern Cape	1·657 [1·172, 2·344]	(0·004) **
First symptom noticed	vaginal bleeding vs pain	1·595 [1·094, 2·325]	(0·015) *
	discharge vs pain	1·418 [0·920, 2·186]	(0·114)
	other vs pain	0·957 [0·532, 1·722]	(0·883)
Number of visits prior to diagnosis		0·740 [0·678, 0·808]	(<0·001) ***
First provider seen	local clinic doctor vs local clinic nurse	1·066 [0·663, 1·712]	(0·792)
	local hospital doctor vs local clinic nurse	0·720 [0·450, 1·150]	(0·169)
	spiritual healer vs local clinic nurse	1·947 [0·252, 15·066]	(0·523)
	unknown vs local clinic nurse	0·380 [0·132, 1·098]	(0·074) +
**Model metrics**			
Number of observations		215	
AIC		1827·3	
BIC		1864·4	
RMSE		1·05	

Abbreviations:

HR: hazard ratio

CI: Confidence interval

AIC: Akaike information criterion; BIC: Bayesian information criterion; RMSE: root mean squared error

*** <0.001, ** 0.001-0.01, * 0.01-0.05, + 0.05-0.1

Supplementary table 3d: Cox regression results for diagnostic interval: cervical cancer, Zimbabwe

Variable	Categories	HR [95% CI]	p-value
Age		1·019 [1·004, 1·035]	(0·014) *
First symptom noticed	vaginal bleeding vs pain	0·580 [0·340, 0·991]	(0·046) *
	discharge vs pain	0·446 [0·251, 0·794]	(0·006) **
	other vs pain	0·518 [0·257, 1·043]	(0·066) +
Number of visits prior to diagnosis		0·733 [0·671, 0·801]	(<0·001) ***
First provider seen	local clinic doctor vs local clinic nurse	0·725 [0·399, 1·318]	(0·292)
	local hospital doctor vs local clinic nurse	0·784 [0·537, 1·144]	(0·206)
	specialist at referral hospital vs local clinic nurse	10·152 [2·317, 44·478]	(0·002) **
	spiritual healer vs local clinic nurse	0·697 [0·349, 1·394]	(0·307)
	unknown vs local clinic nurse	0·343 [0·123, 0·960]	(0·041) *
**Model metrics**			
Number of observations		158	
AIC		1225·2	
BIC		1258·8	
RMSE		1·07	

Abbreviations:

HR: hazard ratio

CI: Confidence interval

AIC: Akaike information criterion; BIC: Bayesian information criterion; RMSE: root mean squared error

*** <0.001, ** 0.001-0.01, * 0.01-0.05, + 0.05-0.1

Supplementary table 3e: Cox regression results for diagnostic interval: colorectal cancer, South Africa

Variable	Categories	HR [95% CI]	p-value
Number of visits prior to diagnosis		0·770 [0·683, 0·868]	(<0·001) ***
First provider seen	local clinic doctor vs local clinic nurse	1·097 [0·508, 2·369]	(0·813)
	local hospital doctor vs local clinic nurse	0·462 [0·193, 1·105]	(0·083) +
	specialist at referral hospital vs local clinic nurse	4·071 [1·141, 14·523]	(0·031) *
	spiritual healer vs local clinic nurse	2·241 [0·278, 18·075]	(0·449)
	unknown vs local clinic nurse	1·196 [0·254, 5·624]	(0·821)
**Model metrics**			
Number of observations		105	
AIC		755·1	
BIC		773·7	
RMSE		0·94	

Abbreviations:

HR: hazard ratio

CI: Confidence interval

AIC: Akaike information criterion; BIC: Bayesian information criterion; RMSE: root mean squared error

*** <0.001, ** 0.001-0.01, * 0.01-0.05, + 0.05-0.1

Appendix 3f: Cox regression results for diagnostic interval: colorectal cancer, Zimbabwe			
**Variable**	**Categories**	**HR [95% CI]**	**p-value**
Relationship status	single vs married/living with a partner	0·526 [0·177, 1·564]	(0·248)
	separated/divorced/widowed vs married/living with a partner	0·524 [0·280, 0·982]	(0·044) *
First symptom noticed	bleeding vs pain	0·737 [0·348, 1·561]	(0·426)
	colorectal habits vs pain	0·255 [0·110, 0·591]	(0·001) **
	other vs pain	0·689 [0·344, 1·379]	(0·293)
Number of visits prior to diagnosis		0·773 [0·684, 0·874]	(<0·001) ***
First provider seen	local clinic doctor vs local clinic nurse	3·040 [1·077, 8·583]	(0·036) *
	local hospital doctor vs local clinic nurse	1·788 [0·879, 3·637]	(0·109)
	specialist at referral hospital vs local clinic nurse	1·364 [0·386, 4·822]	(0·629)
	spiritual healer vs local clinic nurse	0·454 [0·163, 1·265]	(0·131)
	unknown vs local clinic nurse	12·410 [0·974, 158·144]	(0·052) +
**Model metrics**			
Number of observations		71	
AIC		449·3	
BIC		476·4	
RMSE		0·94	

Abbreviations:

HR: hazard ratio

CI: Confidence interval

AIC: Akaike information criterion; BIC: Bayesian information criterion; RMSE: root mean squared error

*** <0.001, ** 0.001-0.01, * 0.01-0.05, + 0.05-0.1
